# Tankyrase Inhibitor for Cardiac Tissue Regeneration: an *In-silico* Approach

**DOI:** 10.22037/ijpr.2021.115367.15339

**Published:** 2021

**Authors:** Faezeh Sadat Hosseini, Arash Amanlou, Massoud Amanlou

**Affiliations:** a *Department of Medicinal Chemistry, Faculty of Pharmacy, Tehran University of Medical Sciences, Tehran, Iran. *; b *Experimental Medicine Research Center, Tehran University of Medical Sciences, Tehran, Iran.*

**Keywords:** Tankyrase inhibitor, Cardiac tissue regeneration, Virtual screening, Molecular dynamic simulation, Wnt/β-catenin signaling

## Abstract

Myocardial infarction causes heart tissue damages; therefore, using non-invasive methods to regenerate the heart tissue could be very helpful. Recent studies claimed that the inhibition of the Wnt signaling could promote cardiac remodeling and induce cardiac regeneration. Therefore, a tankyrase inhibitor to stabilize the AXIN and inhibit the Wnt/β-catenin signaling pathway will induce cardiac regeneration after injury. In this regard, virtual screening procedure, using molecular docking of 9127 FDA and world approved drugs, including herbal medicine, was done over the crystal structures of tankyrase 1 (TNKS1) and tankyrase 2 (TNKS2) catalytic poly (ADP-ribose) polymerase (PARP) domains with PDB ID: 2RF5 and 3KR7, respectively, to find potential small molecule inhibitors to regenerate injured heart tissue. Subsequently, molecular dynamics simulations were done to assess the stability of selected ligands phenothrin and ethyl rosinate in the binding pocket of TNKS1 and TNKS2 for 100 ns, respectively. Both compounds show suitable interaction in their binding pocket. The molecular dynamics simulation results confirm their stability. The binding free energy of complexes was carried out by the MM-PBSA method. ADME properties also indicate the potential of drug-likeness of both compounds. Taking together both drugs may be promising for inducing cardiac regeneration after injury. Nevertheless, clinical approval remains.

## Introduction

Cardiovascular diseases such as myocardial infarction (MI) and heart failure are causes of death globally (1). Because of the heart tissue damages, the therapeutic approaches and approved medications do not have satisfactory results (2). The Surgical procedure to treat heart tissue damage such as heart muscle reconstruction or heart transplantation is just limited to exceptional cases. Therefore, using newer methods such as regenerative medicine can be very helpful (3).

**Figure 1:**
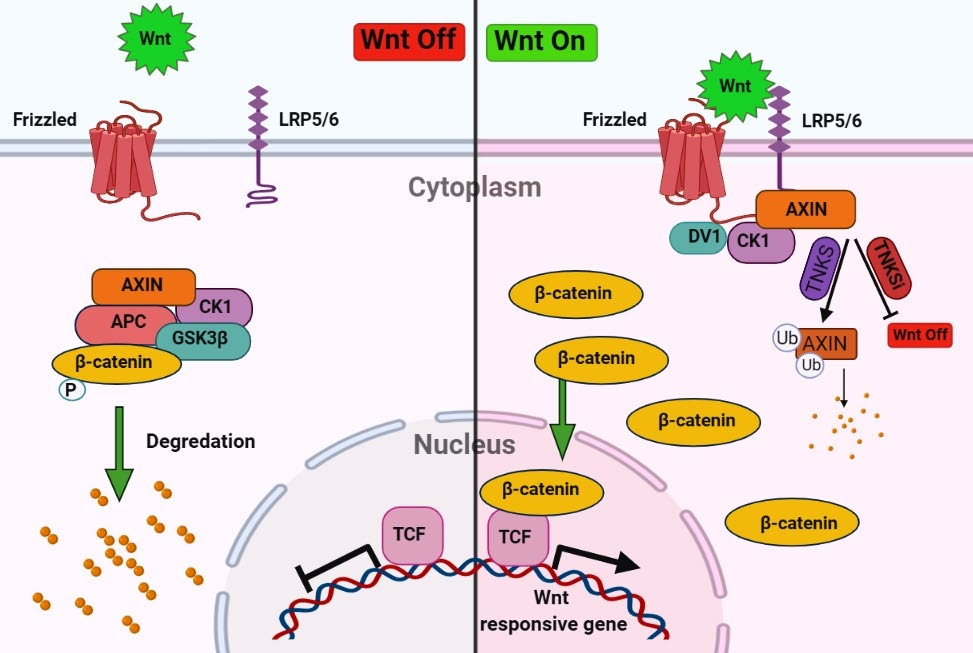
Overview of Wnt/β-catenin signaling pathway. In the absences of Wnt signaling degradation and phosphorylation of β-catenin will occur by the destruction complex, consisting of Axin, casein kinase 1α (CK1α), glycogen synthase kinase 3β (GSK3β), and the tumor suppressor adenomatous polyposis coli (APC) and in the presence of Wnt signaling cytosolic β-catenin translocation to the nucleus and transcript the genes. Tankyrase stabilized the β-catenin degradation by poly(ADP-ribosyl)ation (PARsylation) of Axin. Inhibitors of tankyrase (TNKSi) stabilize the AXIN and inhibit the Wnt/β-catenin signaling pathway

 The tissue repair process is a dynamic cell proliferation and tissue regeneration response to restore normal organ/tissue structure and function (4). There are different mechanisms and mediators involved in cell proliferation and tissue repair. Nevertheless, one of the most critical pathways in the regeneration process is a highly conserved Wnt/β-catenin signaling pathway which plays a fundamental role during embryonic development and differentiation, adult homeostasis, and tissue regeneration after injuries. Besides, the imbalance of Wnt signaling activity leads to various diseases such as colon cancer, hair follicle tumors, and leukemia (5-7). The final output of the Wnt/β-catenin signaling prevents the degradation of cytosolic β-catenin and its translocation to the nucleus and eventually transcription of genes. Whereas the absences of Wnt signaling leading to degradation and phosphorylation of β-catenin by the destruction complex, consisting of Axin, casein kinase 1α (CK1α), glycogen synthase kinase 3β (GSK3β), and the tumor suppressor adenomatous polyposis coli (APC) (8, 9). Tankyrase 1 (TNKS1) and tankyrase 2 (TNKS2) stabilized the β-catenin degradation by poly(ADP-ribosyl)ation (PARsylation) of Axin (Figure 1) (10). Therefore, the tankyrase inhibitor stabilized the Axin by increased destruction of β-catenin and decreased Wnt/β-catenin signaling (11). 

Wnt/β-catenin signaling is crucial for cardiac specification in the early stages of development, but after that, the inhibition is essential for maturation in the adult heart (12). Wnt signaling is silent, but it is activated after cardiac injury (13); therefore, inhibition of the Wnt signaling could promote cardiac remodeling (14) and preserve cardiac function (15) and decrease fibrosis and induce cardiac regeneration (16).

 Due to the importance of tankyrase in Wnt/β-catenin signaling, using TNKS inhibitor in several diseases including cancer (colon, lung, and prostate), cherubism, and systemic sclerosis, Epstein Barr and Herpes simplex viral infections, fibrotic diseases, and others have been investigated before (17-20). 

The Discovery of novel inhibitors of tankyrase to stabilize the AXIN and inhibit the Wnt/β-catenin signaling pathway to induce cardiac regeneration after a cardiac injury has been discussed in this article. In this regard, a virtual screening procedure, using docking of 9127 FDA and world-approved drugs, including herbal medicine and some chemical compound with possible efficacy, was done over crystal structure of TNKS1 and TNKS2 to find potential therapeutic small molecule inhibitors to regenerate injured heart tissue. Due to the diverse biological activities of the signaling pathway Wnt/β-catenin, the present study was designed to test the hypothesis of whether regeneration of heart tissue cells occurs after ischemic injury using tankyrase inhibitors.

## Experimental

*CAVER 3.0.1 software*

Caver software was used to identify the hypothetic tunnel of the active site of the TNKS1 and TNKS2 leading into the key residues (21). Caver parameters were set as follows: maximum probe radius as 9 Å, shell depth 4 Å, and clustering threshold 3.5 Å. 

*Molecular Docking Strategy*

To achieve novel high-affinity tankyrase inhibitors, docking-based virtual screening was performed using Parallelized Open Babel and AutoDock suite Pipeline (POAP) (22). POAP is a GPU integrated tool of Open Babel, AutoDock Vina, AutoDock, and AutoDockZN used for boosted high throughput virtual screening. The crystal structures of Tankyrase 1 and 2 catalytic Poly (ADP-ribose) polymerase (PARP) domains were retrieved from the Protein Data Bank (www.rcsb.org) with PDB ID: 2RF5 and 3KR7, respectively. Due to zinc ion presence in tankyrase structure, the preparation of receptor input files was done using AutoDockZN in POAP (23). 

3D structures of ligands in the structure data file (SDF) format containing 9127 approved drugs, regulated chemicals, and herbal isolates were retrieved from the SWEETLEAD database (24) and converted to PDB format using POAP. 

A 60×60 ×60 Å (x, y, and z) grid box with 0.375 nm spacing for each dimension was centered in the active site and embedded for AutoGrid to prepare grid maps. Dockings were performed using the Lamarckian Genetic Algorithm (LGA) using AutoDockZN with the aid of scripts provided by POAP. Docking parameters were set as follows: initial population=150, the number of Lamarckian jobs =10, the maximum number of energy evaluation=2.5×10^5^, and other parameters were set in their default value. Discovery Studio Visualizer version 17.2 (25) and PyMol version 1.1evel (26) were used to visualize docking results.

*Classical Molecular dynamics simulation*

The complexes of phenothrin with TNKS1 and ethyl rosinate with TNKS2 from the docking results were used as the input file for molecular dynamic simulation. All-atom molecular dynamics simulation was done by GROMACS-2020.3 software package (http://www.gromacs.org) and the latest version of CHARMM36-jul2020 as a force field for 100 ns. The topology files of the ligands were generated by the CGenFF web server (27). The TIP3P water model encompasses the complex in a dodecahedron box. To neutralize the system, proper counter ions were added. The steepest-descent method was used for the energy minimization with a tolerance of 1000 kJ/mol/nm and 50000 minimization steps. The long-range Van der Waals cut-off was 1.2 nm, and the same was set for the long-range electrostatic cut-off of particle mesh Ewald method (PME). The converged system was equilibrated with NVT and NPT ensembles for 100 ps. The Berendsen thermostat and barostat have held the temperature and pressure constant at 300 K and 1 bar. The equilibrated system went through the MD simulation run for 100 ns. The structural deviations of RMSD and RMSF and the number of hydrogen bonds were calculated.

*MM-PBSA calculation*

An important feature of MD simulations and thermodynamic calculations coupling measures the binding free energy of a protein-ligand complex. The combination of Molecular Mechanic / Poisson-Boltzmann Surface Area (MMPBSA) with MD simulation has been reported to successfully calculate the binding free energy of the complex calculated according to the following equation: 

∆G _binding_ = ∆G _(complex) _- ∆G _(Protein) _- ∆G _(Ligand)_

Where ∆G_(complex)_ represents the free energy of complex (protein-ligand), ∆G _(Protein)_ is the free energy of relaxed protein without the ligand, and ∆G_(Ligand)_ is the free energy of the alone ligand after removing it from the protein in the solvent. The total free energy of a complex, protein, or ligand could be calculated from its molecular mechanic’s potential energy plus the energy of solvation. Therefore, the g_mmpbsa compiled for GROMACS 2020.3 was used to perform MM-PBSA calculations through phenothrin’s MD trajectories with TNKS1 and ethyl rosinate with TNKS2 (28).

## Results

Wnt signaling should be blocked to authorized natural heart regeneration; hence, finding potential small molecules inhibitor for one of these pathway components like TNKS could activate heart regeneration after injury. In this regard, virtual screening was performed over approved drugs, regulated chemicals, and herbal isolates from the SWEETLEAD database over TNKS1 and TNKS2. The structures were sorted and ranked based on the docking binding energy (Tables 1 and 2).

*Identification of the Active site*

**Figure 2:**
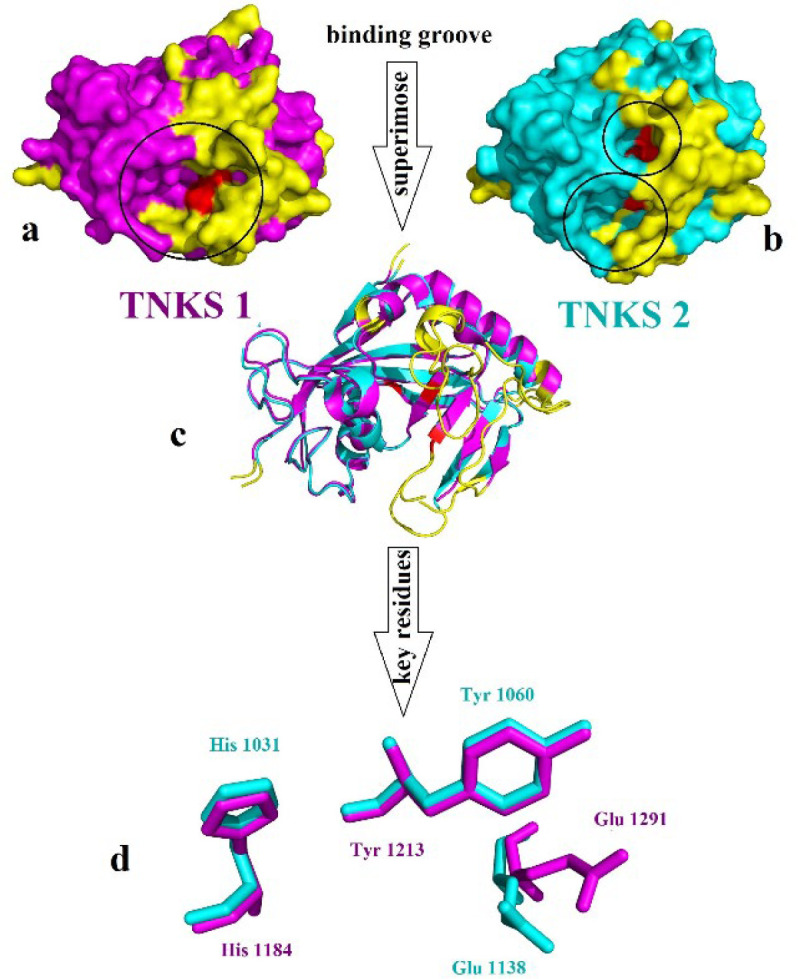
(a) Surface representation of TNKS1 in magenta color, (b) Surface representation of TNKS2 in cyan color the yellow color refers to the differences part of the structure which does not align to each other, red color indicates the place of crucial residues and (c) Alignment of TNKS1 and TNKS2 in cartoon representation (d) Alignment of the crucial residue of the active site of TNKS1 in magenta and TNKS2 in cyan

Tankyrase proteins stabilized the β-catenin during the degradation process with Axin protein (29). Tankyrase belongs to the poly(ADP)-ribose polymerases (PARPs) family, which utilizes NAD^+^ to catalyze ADP-ribose (ADPr) on target proteins. Tankyrase has two isoforms: tankyrase 1 (TNKS1) and tankyrase 2 (TNKS2), also known as ADP-ribosyltransferases ARTD5 and ARTD6, respectively. Tankyrase contains three particular domain, five ankyrin repeat (ANK) domain with regulating role in protein-protein interaction, the sterile alpha motif domain (SAM) for oligomerization located N terminal and the catalytic ADP-ribosyltransferase (ARTD) domain at the C-terminus (30-32). TNKS1 and TNKS2 indicate 85% similarity in amino acid sequences and have 1327 and 1166 residues, respectively. The catalytic domain shares 89% overall sequence identity and contains three conserved central amino acids: His1184, Tyr1213, and Glu1291 in TNKS1, and the residue His1031, Tyr1060, and Glu1138 in TNKS2. Except for the common domain, as noted before, TNKS1 has a special His, Pro, Ser rich (HPS) region in the N-terminus with unknown function Figure 2 (33). 

**Figure 3.**
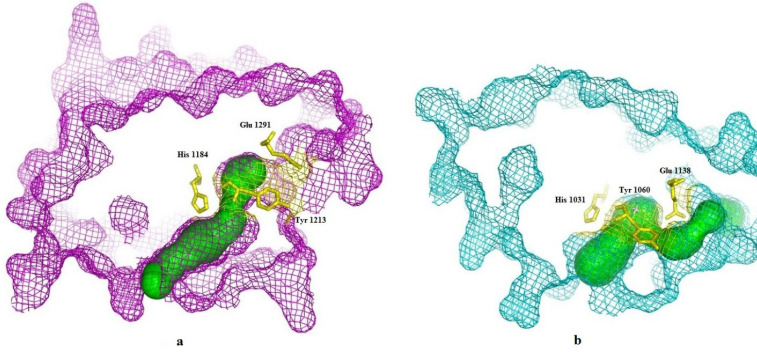
CAVER result and predicted tunnel reached the active site of (a) TNKS1 in magenta and (b) TNKS2 in cyan

CAVER 3.0.1. software was used to predict the possible tunnels of the TNKS1 and TNKS2. The results indicate that for each protein, one tunnel leads into the key residues Figure 3. The tunnel identified by CAVER for TNKS1 is much longer than the predicted tunnel of the TNKS2. Despite the sequence similarity between TNKS1 and TNKS2, the binding groove shapes make a difference in virtual screening results. 

*Docking based virtual screening*

Virtual screening was performed over the 9127 ligands of the SWEETLEAD database over TNKS1 and TNKS2, and the obtained structures were ranked based on the docking binding energy (34). The top ten screened compounds were listed in Tables 1 and 2. Docking binding energy has shown the score ranging from -12.84 to -11.5 kcal/ mol and -11.70 to -11.08 kcal/ mol for TNK1 and TNKS2.

The result indicates that in TNKS1, the ligands with longer carbon side chains and higher volume were placed well in the binging groove, but in TNKS2, the ligands with shorter side chains were placed following the binding track volume were able to fit in the binding groove. Interestingly, on average, the binding groove of TNKS1 indicates more hydrophobic properties than TNKS2, and the compounds with the higher value of miLogP could fit well in the binding groove. 

Three top ligands of each screening were investigated for their interactions. In general, lipophilic interactions and van der Waals interaction play an essential role in binding. The amino acids involved in binding phenothrin and TNKS1 are critical residue Tyr1213 with van der Waals and π-alkyl interactions, Tyr1224 with π-sigma, and π-alkyl interaction. Lots of π-alkyl or alkyl interactions with His1184, Ile1192, Tyr1203, Ile1204, Ala1215, Ile1212, and critical residue Glu1291 is also forming van der Waals interactions. Regarding bornyl trans-cinnamate, hydrogen bond with Ile1204 and π-π stacking with Tyr1224 and several π-alkyl interactions with His1184, Phe1188, Ile1192, Phe1197, His 1201, Tyr1203, and Ile1212 were observed. Eletriptan indicates four hydrogen bonds with crucial residue His1184, Gly1185, His1201, and Gly1211. Ile1212 form π-σ and π-π stacking interactions were seen with Tyr1224 and π-alkyl interactions with Ala1215 and Lys1220. 

**Figure 4:**
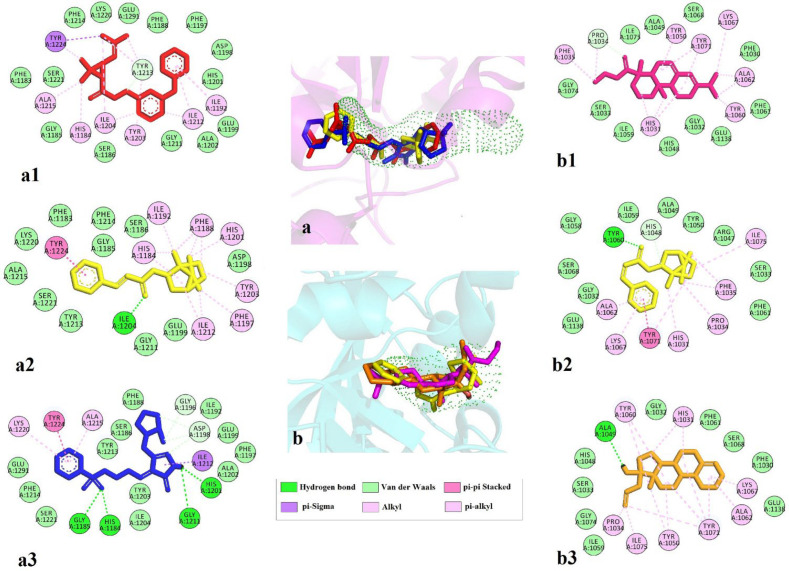
2D interactions of top three ligands of docking result. (a) Tankyrase 1 and predicted tunnel by caver and the superimpose of the top-ranked three ligands phenothrin (a1, red), bornyl trans-cinnamate (a2, yellow), and eletriptan (a3, blue); (b) Tankyrase 2 and predicted tunnel by caver and the superimpose of the top-ranked three ligands ethyl rosinate (b1, pink) bornyl trans-cinnamate (b2, yellow), and allylestrenol (b3, orange)

TNK2 interact with ethyl rosinate (Abietic acid ethyl ester) with van der Waals interaction of Pro1034 and several and π-alkyl interactions with key residue His1031, Phe1035, Tyr1050, essential residue Tyr1060, Ala1062, Lys1067, and Tyr1071. Bornyl trans-cinnamate forms hydrogen bond with essential residue Tyr1060, π-π stacking with Tyr1071, and lots of π-alkyl interactions with key residue His1031, Pro1034, Phe1035, Ala1062, Lys1067, and Ile1075. Allylestrenol indicates hydrogen bond with Ala 1049 and several π-alkyl interactions with critical residue His1031, Pro1034, Tyr1050, essential residue Tyr1060, Ala1062, Lys1067, and Tyr1071 Figure 4.

*Molecular dynamics simulation*

**Figure 5:**
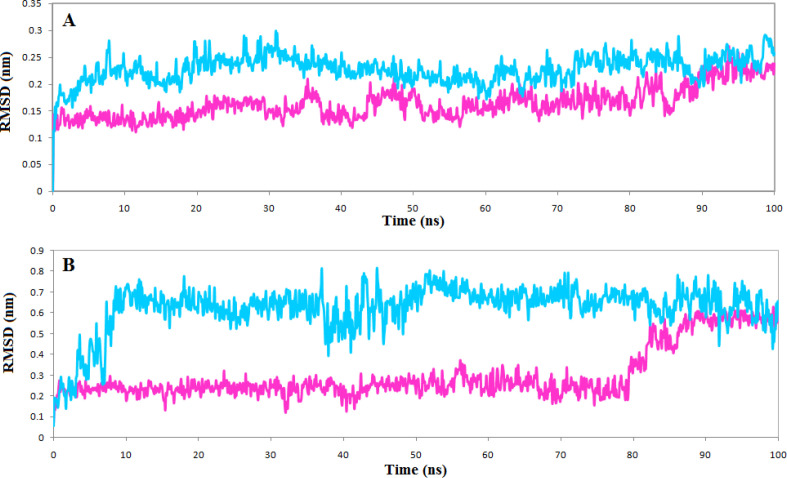
(A) Backbone RMSD plots of the Tankyrase 1 (magenta) and Tankyrase 2 (cyan) (B) Backbone RMSD plots of the ligands phenothrin (magenta) and ethyl rosinate (cyan)

The top compounds of each list, phenothrin, and ethyl rosinate in complexes with the TNKS1 and TNKS2, were further evaluated for stability by molecular dynamics simulations for 100 ns, and the structural deviations were calculated. The backbone atom deviation was applied to determine the stability of both complexes. RMSD plot shows that both complexes stabilized after 20 ns, but the TNKS1 in complex with phenothrin indicated slight deviations and reached a steady station faster Fig 5A. The same trend is seen for the RMSD plot of ligand, and the phenothrin reached a stable state very soon and maintained stability in the entire simulation period Figure 5B. The average RMSD value of phenothrin in complex with TNKS1 was 0.2 nm, close to the obtained average RMSD value of 0.136 nm for the ligand ZINC28852318 (N-(4-morpholinophenyl)-3-(3-oxopiperazin-1-yl)propanamide) reported recently (35). 

**Figure 6:**
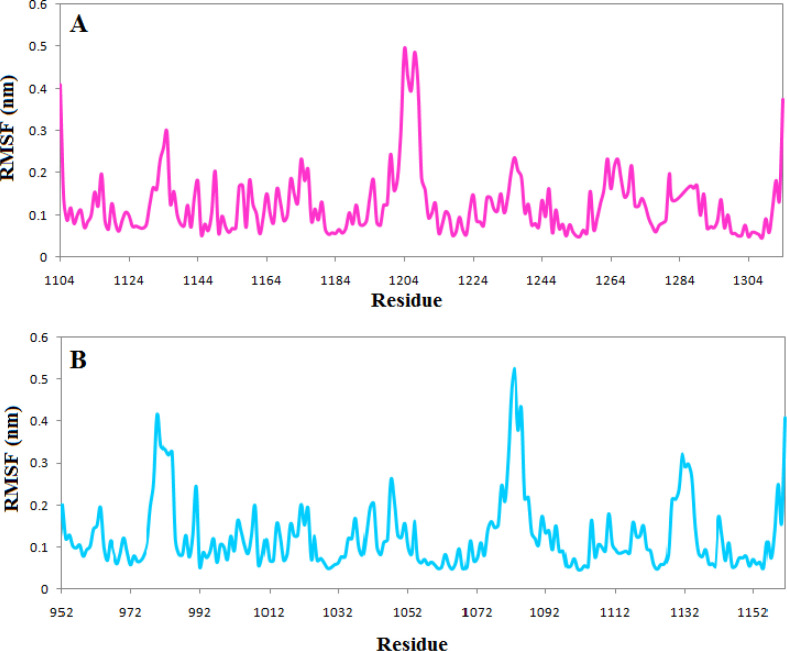
RMSF plot of the (A) Tankyrase 1 in complex with phenothrin (magenta) (B) Tankyrase 2 in complex with ethyl rosinate (cyan)

RMSF profile indicates that the active site’s vital amino acids do not have flexibility in both complexes, and the occupation of the ligands reduced the fluctuation of the active site. However, just as the RMSD plot showed that the complexes of TNKS1 with phenothrin had stabilized earlier, there is less fluctuation in the RMSF plot indicating the ligand’s ability to deprive the protein of flexibility Figure 6

**Figure 7:**
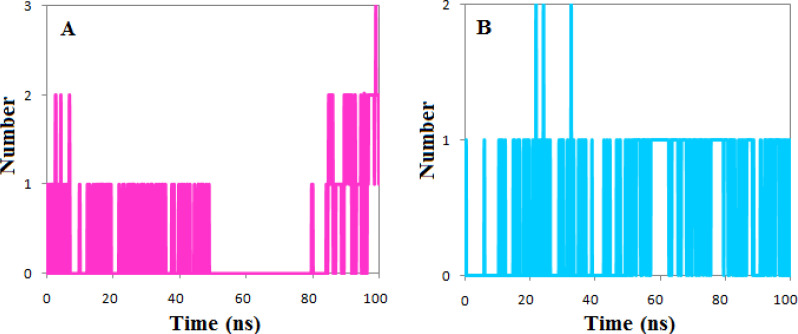
The number of the hydrogen bond between (A) Tankyrase 1 and phenothrin (magenta) (B) Tankyrase 2 and ethyl rosinate (cyan)

The calculated number of the hydrogen bonds shows that both ligands had at least one hydrogen bond during the simulation period except the 30 ns of simulation of the complexes of phenothrin. However, the number of hydrogen bonds has reached three at the end of simulations, but the ethyl rosinate established only one hydrogen bond during the simulation (Figure 7).

*MM-PBSA binding free energy calculations*

G_mmpbsa package was used to calculate the binding free energy, which is an important indicator that accounts for the potential affinity of the ligand to the receptor. In general, complexes with lower binding free energy can be considered more stable, and their ligands are expected to have a higher inhibitory effect and potency. Each conformation of the MD simulation trajectory was used to calculate the binding free energies by the MM-PBSA method. 

Accordingly, the binding free energy for the two complexes of phenothrin in complexes with the TNKS1 and the ethyl rosinate in complexes with the TNKS2 were employed over the MmPbSaStat.py python script (28). The total free binding energy of each complex component, i.e., the energy of the complex, protein, and ligand, was calculated by this script. 

Furthermore, the cumulative sum of the molecular mechanic’s potential energy in a vacuum and the free energy of solvation includes the polar (electrostatic) and nonpolar (non-electrostatic) solvation energy are the components of free binding energy calculations. The nonpolar solvation energy was usually calculated by the model of solvent-accessible surface area (SASA). All types of energies and the value of standard deviation were calculated by the g_mmpbsa package. Then to obtain the average of the free energy of each component, they were summed together. Finally, the total binding free energy is obtained by subtracting the receptor and ligand’s total free energy from the complex’s total free energy. Table 3 summarizes the interaction energies and the binding free energy for the two complexes.

The results of MM-PBSA calculation of the Free Gibbs energy of the phenothrin in complexes with the TNKS1 indicate the slightly higher binding affinities to the TNKS1 than the ethyl rosinate in complexes with the TNKS2. Phenothrin in complexes with the TNKS1 was better than ethyl rosinate in complexes with the TNKS2 in all the calculated energy formats except (electrostatic energy). Phenothrin average binding free energy reached –115.18 kJ/mol, while ethyl rosinate average binding free energy reached –111.87 kJ/mol, which is similar to the trend of docking results in binding energy. The overall results of the dynamic simulations supported our design concept and validated the entire virtual screening approach; they also emphasized the potential inhibitory effect of phenothrin on TNKS1.

*ADME properties*

SwissADME server was used to determine the drug-likeness of selected compounds phenothrin and ethyl rosinate (36). As Table 4 shows, both compounds fulfill the criteria of Lipinski’s Rule of five (37) in terms of hydrogen bond donors (HBD) and hydrogen bond acceptors (HBA) logP and molecular weight (MW). The solubility class of phenothrin is poorly soluble, but ethyl rosinate indicates moderate solubility. Both ligands show topological polar surface area (TPSA) values lower than 140 Å^2^**,** which means good cell permeability. Therefore, both compounds indicate the potential of drug-likeness properties.

## Discussion

The role of the tankyrase enzyme has been seen in many conditions such as cancer, Wnt/β-catenin signaling, HSV replication, cardiomyocytes, telomeric dysfunction, mitosis, Cherubism, and many other diseases. Therefore, the design and development of the inhibitor for this target are in high demand. The varieties of chemical structures have been known as TNKS inhibitors. 

The flavones scaffold, known as a health enhancer, has been observed as TNKS inhibitors with an IC_50_ 6 nM for compound **1** (38). The docking study indicates that flavon core interacts with TNKS2 by forming the hydrogen bond from the carbonyl oxygen atom with residues GLY1032 and SER1068, also the pi interactions with residues HIS1031 and TYR1071 (39). 

Compound **2,** a triazole analog, inhibits the TNKS with an IC50 value of 75 nM (40). Molecular docking shows that the residue MET1207 and LYS1195 of TNKS1 interact with the nitrile moiety and the oxygen atom of the sulfonyl group of compound **2** by forming the hydrogen bond. Furthermore, residue SER1186 interacts with 1,2,4-triazole nitrogen. Compound **2 **interacts with TNKS2 by forming the hydrogen bond between the oxygen atom of the sulfonyl group and residue MET1054. TYR1060 forms p-sulfur interaction with interacts with sulfonyl group (39). 

**Figure 8:**
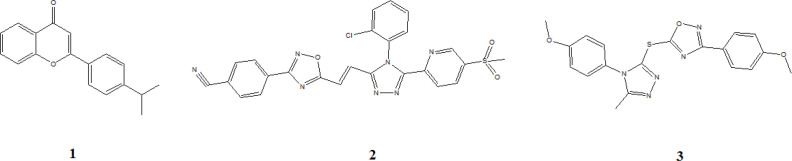
Chemical structure of tankyrase inhibitors

Compound **3** as an oxadiazoles analogs displayed the inhibition range for TNKS2 and TNKS1 in 175 nM and 559 nM, respectively (41). Molecular docking shows compound **3** form two hydrogen bonds with residues LYS1195 and TYR1213 of TNKS1. However, residues MET1054, GLY1127, HIS1048, and GLU1138 of TNKS2 form carbon-hydrogen bonding interaction with compound **3**. Besides, many other chemical scaffolds such as oxazolidinone, tetrazoloquinoxaline, tetrahydro-naphthyridin-5-ones, and isoquinoline-1(2H)-one are available as tankyrase inhibitor (39) (Figure 8).

 Here we hypothesize that using tankyrase inhibitors will inhibit the Wnt/β-catenin signaling and help the regeneration of heart tissue cells after injury. Our docking study combined with molecular dynamic simulation indicates significant results for new structural inhibitors of phenothrin and ethyl rosinate for tankyrase enzyme, which could be a promising option for cardiac regeneration. 

**Table 1:** Top ten potential inhibitors from virtual screening of SWEETLEAD databases over Tankyrase 1

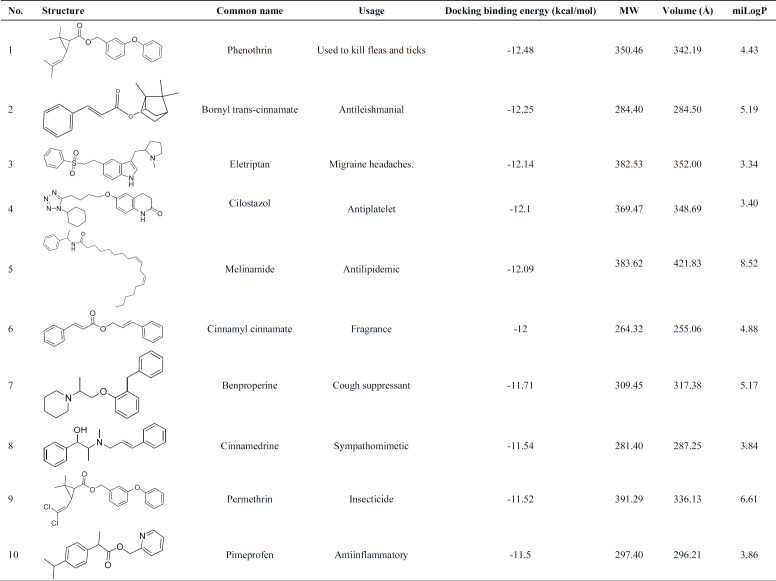

**Table 2:** Top ten potential inhibitors from virtual screening of SWEETLEAD databases over Tankyrase 2.

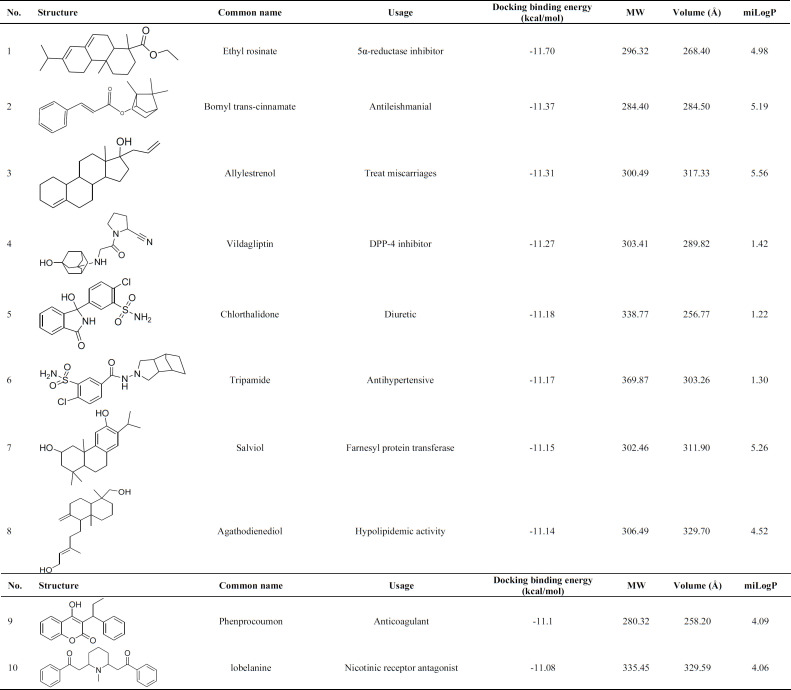

**Table 3:** The calculated interaction energies and the binding free energy for phenothrin and ethyl rosinate complexes with tankyrase 1 and tankyrase 2 catalytic poly (ADP-ribose) polymerase (PARP) domains

**Complex**	**ΔE **_binding (kJ/mol)_	**ΔE **_Electrostatic _ _(kJ/mol)_	**ΔE **_Vander Waal_ _(kJ/mol)_	**ΔE **_polar solvation (kJ/mol)_	**SASA **_(kJ/mol)_
Phenothrin	–115.18 ± 14.51	–10.62 ± 8.33	–203.05 ± 20.59	121.11 ± 19.33	–22.61 ± 1.12
Ethyl rosinate	–111.87 ± 12.55	–17.93 ± 8.82	–159.27 ± 11.62	83.45 ± 15.44	–18.123 ± 1.04

**Table 4:** ADME properties of selected compounds

**Entry**	**H-bond acceptors**	**H-bond donors**	**TPSA** **(Å**^2^**)**	**miLOGP**	**Lipinski**	**Solubility**
Phenothrin	3	0	35.53	4.43	Yes	Poorly soluble
Ethyl rosinate	2	0	26.3	4.98	Yes	Moderately soluble

## Conclusion

The heart tissue damages after the myocardial infarction are one of the causes of mortality. One way to regenerate the heart tissue after the injury is the inhibition of the Wnt signaling. Therefore, we use the virtual screening method combined with molecular dynamic simulations to find the potential inhibitor for TNKS; thus, stabilizing the AXIN and then inhibiting the Wnt/β-catenin signaling pathway will occur. The result indicates that phenothrin and ethyl rosinate could fit well into the binding pocket of TNKS1 and TNKS2, respectively, and both drugs may be promising for inducing cardiac regeneration after injury. Nevertheless, clinical approval remains. 

## Declaration of Competing Interest

The authors declare that they have no known competing financial interests or personal relationships that could have influenced the work reported in this paper.
